# *PPARGC1A* rs3736265 G>A polymorphism is associated with decreased risk of type 2 diabetes mellitus and fasting plasma glucose level

**DOI:** 10.18632/oncotarget.16307

**Published:** 2017-03-17

**Authors:** Li Zhu, Qiuyu Huang, Zhiqiang Xie, Mingqiang Kang, Hao Ding, Boyang Chen, Yu Chen, Chao Liu, Yafeng Wang, Weifeng Tang

**Affiliations:** ^1^ Department of Nephrology, Affiliated People's Hospital of Jiangsu University, Zhenjiang, Jiangsu Province, China; ^2^ Department of Cardiac Surgery, Fujian Medical University Union Hospital, Fuzhou, Fujian Province, China; ^3^ Department of Clinical Laboratory, Fujian Medical University Union Hospital, Fuzhou, Fujian Province, China; ^4^ Department of Thoracic Surgery, Fujian Medical University Union Hospital, Fuzhou, Fujian Province, China; ^5^ Department of Respiratory Disease, Affiliated People's Hospital of Jiangsu University, Zhenjiang, Jiangsu Province, China; ^6^ Department of Medical Oncology, Fujian Provincial Cancer Hospital, Fujian Medical University Cancer Hospital, Fuzhou, Fujian Province, China; ^7^ Department of Cardiothoracic Surgery, Affiliated People's Hospital of Jiangsu University, Zhenjiang, Jiangsu Province, China; ^8^ Department of Cardiology, The People's Hospital of Xishuangbanna Dai Autonomous Prefecture, Jinghong, Yunnan Province, China

**Keywords:** PPARG, PPARGC1A, PPARGC1B, polymorphism, type 2 diabetes mellitus

## Abstract

It has been reported that peroxisome proliferator-activated receptor gamma (PPARG) and peroxisome proliferator-activated receptor gamma co-activator 1 (PPARGC1) family (e.g. PPARGC1A and PPARGC1B) are key agents in the development and pathophysiology of type 2 diabetes mellitus (T2DM). In this study, we designed a case-control study and selected *PPARG* rs1801282 C>G, *PPARG* rs3856806 C>T, *PPARGC1A* rs8192678 C>T, *PPARGC1A* rs2970847 C>T, *PPARGC1A* rs3736265 G>A, *PPARGC1B* rs7732671 G>C and *PPARGC1B* rs17572019 G>A polymorphisms to assess the relationship between these polymorphisms and T2DM using the SNPscan method. A total of 502 T2DM patients and 784 non-diabetic controls were enrolled. We found that *PPARGC1A* rs3736265 G>A polymorphism was correlated with a borderline decreased susceptibility of T2DM. In a subgroup analysis by age, sex, alcohol use, smoking status and body mass index, a significantly decreased risk of T2DM in <65 years and female groups was found. Haplotype comparison analysis indicated that CTTCGGG and CTCTGGG haplotypes with the order of *PPARG* rs1801282 C>G, *PPARG* rs3856806 C>T, *PPARGC1A* rs8192678 C>T, *PPARGC1A* rs2970847 C>T, *PPARGC1A* rs3736265 G>A, *PPARGC1B* rs7732671 G>C and *PPARGC1B* rs17572019 G>A polymorphisms in gene position significantly increased the risk of T2DM. However, CCCCACA haplotype conferred a decreased risk to T2DM. We also found that *PPARGC1A* rs3736265 A allele decreased the level of fasting plasma glucose (FPG), while increased the level of Triglyceride. In conclusion, Our findings suggest that variants of *PPARGC1A* rs3736265 G>A polymorphism decrease the level of FPG, improving the expectation of study in individual's prevention strategies to T2DM.

## INTRODUCTION

Type 2 diabetes mellitus (T2DM) is a most common form of diabetes and is a major public health threat. It is estimated that the prevalence of T2DM in Chinese adult is about 11.6% [1]. T2DM appears to be increasing dramatically worldwide and the vital susceptibility factors contributing to this phenomenon are poor diet, obesity, and sedentary lifestyle [2, 3]. It is reported that both environmental risk factors and genetic components play important roles in the etiology and pathogenesis of T2DM.

T2DM, a complex metabolic disorder, is characterized by hyperglycemia with varying degrees of impaired insulin secretion and insulin resistance (IR) as a result of pancreatic β-cell dysfunction. Imbalance of energy metabolism is considered to be one of the important pathophysiological changes in T2DM, a disease which is also characterised by IR and hyperglycaemia. Accumulating evidence suggests that dysfunction of adipose tissue is contributing to the development of IR and T2DM. Obesity represents a situation of increased fat accumulation, whereas lipodystrophy indicates a situation in which the capacity of retaining lipid in adipocytes is impaired, and then prevents the accumulation of fat. In these situations, the ability of retaining lipid in adipose tissue is impaired, leading to lipotoxicity and consequently developing peripheral IR [4]. There is also evidence that saturated fatty acids are stored in non-adipocyte, decrease glucose conversion into glycogen, and result in cellular damage as a sequence of their lipotoxicity [5]. The lipotoxicity, in the β-cell, has also been considered to contribute to the etiology and pathology of T2DM [6].

In view of that, a number of studies highlighted the vital roles of energy metabolism relative genetics in determining T2DM risk; understanding single nucleotide polymorphisms (SNPs) correlated with T2DM susceptibility may be helpful for providing personalized diagnosis and prevention. The peroxisome proliferator-activated receptor gamma (PPARG), an important transcription factor, keep the balance of energy metabolism by promoting either energy dissipation or energy deposition [7]. Recent studies reported that *PPARG* gene was correlated with higher risk to diabetes [8] and the G allele of the rs1801282 C>G polymorphism in *PPARG* gene was associated with T2DM rsk in a genome-wide association study (GWAS) [9] and has been replicated in some case-control studies; however, other studes found no association between this polymorphism and T2DM [10–12]. The peroxisome proliferator-activated receptor gamma co-activator 1 (PPARGC1) family (e.g. PPARGC1A, PPARGC1B) has been considered as a vital regulator of fatty acid oxidation, gluconeogenesis and adaptive thermogenesis [13]. Of late, several studies explored the association between *PPARGC1A* polymorphisms and risk of T2DM. Results of a pooled-analysis suggested that *PPARGC1A* rs8192678G>A and rs2970847C>T polymorphisms were associated with the increased risk of T2DM in the Indian population [14, 15]. However, in these studies, the number of eligible publications and included subjects was limited and the power might be insufficient. Previous study reported that *PPARGC1B* rs7732671G>C and rs17572019G>A variants were associated with the decreased risk of obesity [16]. Thus, they may alter the risk of T2DM. Therefore, in this study, we designed a hospital-based case-control study and selected *PPARG* rs1801282 C>G, *PPARG* rs3856806 C>T, *PPARGC1A* rs8192678 C>T, *PPARGC1A* rs2970847 C>T, *PPARGC1A* rs3736265 G>A, *PPARGC1B* rs7732671 G>C and *PPARGC1B* rs17572019 G>A polymorphisms to assess the relationship between these SNPs and T2DM in an Eastern Chinese Han population using the SNPscan method.

## RESULTS

### Baseline characteristics

The anthropometric data, biochemistry characteristics, demographics and risk factors of all participants are listed in Table 1. As shown in Table 1, the mean ± SD of height, weight, BMI, FPG, total cholesterol, triglyceride, HDL-C and LDL-C levels was significantly higher in the T2DM group compared with non-diabetic normal controls (*P* < 0.05). However, the mean ± SD of systolic pressure and diastolic pressure was not significant. Additionally, Table 1 showed that the present study was fully matched by age, gender, alcohol use and smoking status. The primary information of *PPARG* rs1801282 C>G, *PPARG* rs3856806 C>T, *PPARGC1A* rs8192678 C>T, *PPARGC1A* rs2970847 C>T, *PPARGC1A* rs3736265 G>A, *PPARGC1B* rs7732671 G>C and *PPARGC1B* rs17572019 G>A polymorphisms is showed in Table 2. For these SNPs, the genotyping success rate was more than 99% in all samples. Minor allele frequency (MAF) and HWE in controls are summarized in Table 2.

**Table 1:** Distribution of selected demographic variables and risk factors in type 2 diabetes cases and controls

Variable	Cases (n=502)		Controls (n=782)		*P*^a^
	n	%	n	%	
Age (years)	65.20 (±9.51)		64.67 (±9.80)		0.347
Age (years)					0.113
< 65	227	45.22	389	49.74	
≥ 65	275	54.78	393	50.26	
Sex					0.819
Male	332	66.14	522	66.75	
Female	170	33.86	260	33.25	
Smoking status					0.264
Never	333	66.33	542	69.31	
Ever	169	33.67	240	30.69	
Alcohol use					0.263
Never	453	90.24	690	88.24	
Ever	49	9.76	92	11.76	
Height (m)	1.68 (±0.08)		1.66 (±0.07)		**0.015**
Weight (kg)	67.63 (±11.42)		64.62 (±9.96)		**<0.001**
BMI (kg/m^2^)	24.95 (±3.64)		23.51 (±2.94)		**<0.001**
BMI (kg/m^2^)					**<0.001**
< 24	210		436		
≥ 24	292		346		
Systolic pressure (mmHg)	135.08 (±17.83)		134.02 (±17.71)		0.297
Diastolic pressure (mmHg)	79.79 (±10.35)		80.06 (±10.02)		0.649
FPG (mmol/L)	8.08 (±2.76)		5.13 (±0.49)		**<0.001**
Total cholesterol (mmol/L)	4.61 (±1.24)		4.88 (±1.02)		**<0.001**
Triglyceride (mmol/L)	1.74 (±1.14)		1.55 (±0.96)		0.001
HDL-C (mmol/L)	1.13 (±0.37)		1.30 (±0.37)		**<0.001**
LDL-C (mmol/L)	3.00 (±1.07)		3.14 (±0.82)		0.010

^a^ Two-sided *x*^2^ test and student t test; Bold values are statistically significant (*P* <0.05); BMI, body mass index; FPG, fasting plasma glucose; HDL-C, high-density lipoprotein cholesterol; LDL-C, low-density lipoprotein cholesterol.

**Table 2:** Primary information for *PPARG* rs1801282 C>G, *PPARG* rs3856806 C>T, *PPARGC1A* rs8192678 C>T, *PPARGC1A* rs2970847 C>T, *PPARGC1A* rs3736265 G>A, *PPARGC1B* rs7732671 G>C and *PPARGC1B* rs17572019 G>A polymorphisms

Genotyped SNPs	*PPARG* rs1801282 C>G	*PPARG* rs3856806 C>T	*PPARGC1A* rs8192678 C>T	*PPARGC1A* rs2970847 C>T	*PPARGC1A* rs3736265 G>A	*PPARGC1B* rs7732671 G>C	*PPARGC1B* rs17572019 G>A
Chromosome	3	3	4	4	4	5	5
Function	missense	coding-synonymous	missense	coding-synonymous	missense	missense	missense
Chr Pos (NCBI Build 37)	12393125	12475557	23815662	23815924	23814707	149212243	149212471
Regulome DB Score^a^	—	2b	6	—	6	5	5
MAF^b^ for Chinese in database	0.07	0.25	0.35	0.28	0.23	0.09	0.07
MAF in our controls (n = 782)	0.05	0.22	0.44	0.21	0.16	0.06	0.06
*P* value for HWE^c^ test in our controls	0.973	0.381	0.850	0.281	0.064	0.693	0.305
Genotyping method	SNPscan	SNPscan	SNPscan	SNPscan	SNPscan	SNPscan	SNPscan
% Genotyping value	99.61%	99.61%	99.61%	99.61%	99.38%	99.61%	99.61%

^a^ http://www.regulomedb.org/;

^b^ MAF: minor allele frequency;

^c^ HWE: Hardy–Weinberg equilibrium;

### Association of PPARG rs1801282 C>G, PPARG rs3856806 C>T, PPARGC1A rs8192678 C>T, PPARGC1A rs2970847 C>T, PPARGC1A rs3736265 G>A, PPARGC1B rs7732671 G>C and PPARGC1B rs17572019 G>A polymorphisms with T2DM

The genotype distributions of *PPARG* rs1801282 C>G, *PPARG* rs3856806 C>T, *PPARGC1A* rs8192678 C>T, *PPARGC1A* rs2970847 C>T, *PPARGC1A* rs3736265 G>A, *PPARGC1B* rs7732671 G>C and *PPARGC1B* rs17572019 G>A polymorphisms are listed in Table 3. The genotype distributions of these polymorphisms in controls were suggested to be in HWE. In the analysis of *PPARGC1A* rs3736265 G>A polymorphism, differences in the frequency distribution of the GA/AA genotypes compared with the GG genotype and GA genotype compared with the GG genotype between T2DM patients and non-diabetic controls were found [GA+AA vs. GG: crude odds ratio (OR) = 0.76, 95% confidence interval (CI) = 0.59–0.99, *P* = 0.041 and GA vs. GG: crude OR = 0.76, 95% CI = 0.58–1.00, *P* = 0.049 (Table 3)]. However, *PPARG* rs1801282 C>G, *PPARG* rs3856806 C>T, *PPARGC1A* rs8192678 C>T, *PPARGC1A* rs2970847 C>T, *PPARGC1B* rs7732671 G>C and *PPARGC1B* rs17572019 G>A polymorphisms was not associated with T2DM susceptibility (Table 3). In two genetic models, logistic regression analysis demonstrated that *PPARGC1A* rs3736265 G>A polymorphism was associated with a borderline statistically risk of T2DM. When the *PPARGC1A* rs3736265 GG genotypes were used as the reference group, the GA/AA and GA genotype was correlated with a borderline statistically decreased susceptibility of T2DM [GA+AA vs. GG: adjusted OR = 0.77, 95% CI = 0.59–1.00, *P* = 0.050 and GA vs. GG: adjusted OR = 0.76, 95% CI = 0.58–1.00, *P* = 0.053 (Table 3)].

**Table 3:** Logistic regression analyses of associations between *PPARG* rs1801282 C>G, *PPARG* rs3856806 C>T, *PPARGC1A* rs8192678 C>T, *PPARGC1A* rs2970847 C>T, *PPARGC1A* rs3736265 G>A, *PPARGC1B* rs7732671 G>C and *PPARGC1B* rs17572019 G>A polymorphisms and risk of type 2 diabetes

Genotype	Cases(n=502)		Controls(n=782)		Crude OR (95%CI)	*P*	Adjusted OR ^a^ (95%CI)	*P*
	n	%	n	%				
*PPARG* rs1801282 C>G								
CC	457	91.95	704	90.03	1.00		1.00	
CG	40	8.05	76	9.72	0.80 (0.54-1.20)	0.280	0.76(0.50-1.14)	0.179
GG	0	0	2	0.26	-	-	-	-
GC+GG	40	8.05	78	9.98	0.79 (0.53-1.18)	0.247	0.75 (0.50-1.12)	0.164
CC+GC	497	100	780	99.75	1.00		1.00	
GG	0	0	2	0.26	-	-	-	-
G allele	40	4.02	80	5.12				
*PPARG* rs3856806 C>T								
CC	278	55.94	474	60.61	1.00		1.00	
CT	196	39.44	275	35.17	1.19 (0.94-1.51)	0.140	1.17(0.92-1.48)	0.204
TT	23	4.63	33	4.22	1.17 (0.67-2.03)	0.583	1.19 (0.68-2.08)	0.541
CT+TT	219	44.06	308	39.39	1.21 (0.97-1.52)	0.098	1.19 (0.94-1.50)	0.140
CC+CT	474	95.37	749	95.78	1.00		1.00	
TT	23	4.63	33	4.22	1.10 (064-1.90)	0.728	1.14 (0.65-1.97)	0.653
T allele	242	24.35	341	21.80				
*PPARGC1A* rs8192678 C>T								
CC	138	27.77	250	31.97	1.00		1.00	
CT	251	50.50	382	48.85	1.15 (0.89-1.49)	0.296	1.12 (0.86-1.46)	0.402
TT	108	21.73	150	19.18	1.26 (0.91-1.74)	0.161	1.22 (0.88-1.69)	0.240
CT+TT	269	54.12	532	68.03	1.22 (0.96-1.57)	0.111	1.19 (0.93-1.1.53)	0.178
CC+CT	389	78.27	632	80.82	1.00		1.00	
TT	108	21.73	150	19.18	1.17 (0.89-1.54)	0.269	1.15(0.87-1.52)	0.334
T allele	467	46.98	682	43.61				
*PPARGC1A* rs2970847 C>T								
CC	310	62.37	485	62.02	1.00		1.00	
CT	160	32.19	268	34.27	0.92 (0.72-1.17)	0.495	0.93 (0.73-1.19)	0.582
TT	27	5.43	29	3.71	1.43 (0.83-2.47)	0.194	1.50 (0.87-2.60)	0.148
CT+TT	187	37.63	297	37.98	0.99 (0.78-1.24)	0.899	1.00 (0.79-1.27)	0.979
CC+CT	470	94.57	753	96.29	1.00		1.00	
TT	27	5.43	29	3.71	1.49 (0.87-2.55)	0.144	1.55(0.90-2.68)	0.112
T allele	214	21.53	326	20.84				
*PPARGC1A* rs3736265 G>A								
GG	380	76.61	557	71.41	1.00			
GA	103	20.77	196	25.13	**0.76(0.58-1.00)**	**0.049**	0.76(0.58-1.00)	0.053
AA	13	2.62	27	3.46	0.70(0.36-1.37)	0.295	0.74(0.37-1.46)	0.378
GA + AA	116	23.39	223	28.59	**0.76(0.59-0.99)**	**0.041**	0.77(0.59-1.00)	0.050
GG+GA	483	97.38	753	96.54	1.00		1.00	
AA	13	2.62	27	3.46	0.75(0.38-1.47)	0.403	0.79(0.40-1.56)	0.494
A allele	129	13.00	250	15.98				
*PPARGC1B* rs7732671 G>C								
GG	435	87.53	698	89.26	1.00		1.00	
GC	61	12.27	81	10.36	1.20(0.84-1.70)	0.323	1.20(0.84-1.72)	0.314
CC	1	0.20	3	0.38	0.53(0.06-5.10)	0.582	0.48(0.05-4.71)	0.527
GC+CC	62	12.47	84	10.74	1.18(0.84-1.68)	0.342	1.19(0.83-1.69)	0.341
GG+GC	496	99.80	779	99.62	1.00		1.00	
CC	1	0.20	3	0.38	0.52(0.05-5.05)	0.576	0.47(0.05-4.66)	0.520
C allele	63	6.34	87	5.56				
*PPARGC1B* rs17572019 G>A								
GG	435	87.53	698	89.26	1.00			
GA	60	12.07	80	10.23	1.19(0.83-1.70)	0.338	1.19(0.83-1.71)	0.338
AA	2	0.40	4	0.51	0.79(0.15-4.35)	0.790	0.81(0.14-4.52)	0.808
GA+AA	62	12.47	84	10.74	1.18(0.84-1.68)	0.343	1.19(0.83-1.69)	0.341
GG+GA	495	99.60	778	99.49	1.00			
AA	2	0.40	4	0.51	0.79(0.14-4.31)	0.781	0.80(0.14-4.49)	0.80
A allele	64	6.44	88	5.63				

^a^ Adjusted for age, sex, smoking status, alcohol use and BMI status.

Bold values are statistically significant (*P* <0.05).

### Association of PPARGC1A rs3736265 G>A polymorphism with T2DM in Different Stratification Groups

Table 4 showed the genotype frequencies of *PPARGC1A* rs3736265 G>A polymorphism in the stratified analysis based on age, gender, alcohol use, smoking status and BMI. In female group, after adjustment for age, alcohol use, smoking status and BMI by logistic regression analysis, the GA/AA and GA genotypes of *PPARGC1A* rs3736265 G>A polymorphism were associated with a significantly decreased risk of T2DM compared with the GG genotype [GA+AA vs. GG: adjusted OR = 0.46, 95% CI 0.28–0.74, P = 0.001 and GA vs. GG: adjusted OR = 0.45, 95% CI = 0.28–0.74, *P* = 0.002 (Table 4)]. In <65 years group, after adjustment for gender, alcohol use, smoking status and BMI by logistic regression analysis, the GA/AA and GA genotypes of *PPARGC1A* rs3736265 G>A polymorphism were also associated with a significantly decreased risk of T2DM compared with the GG genotype [GA+AA vs. GG: adjusted OR = 0.67, 95% CI 0.46–0.98, P = 0.038 and GA vs. GG: adjusted OR = 0.60, 95% CI = 0.40–0.90, *P* = 0.013 (Table 4)]. However, in other groups, there was no correlation between *PPARGC1A* rs3736265 G>A polymorphism and the risk of T2DM (*P* > 0.05; Table 4).

**Table 4:** Stratified analyses between *PPARGC1A* rs3736265 G>A polymorphism and type 2 diabetes risk by sex, age, smoking status, alcohol consumption and BMI

Variable	(case/control)^a^				Adjusted OR^b^ (95% CI); *P*				
	GG	GA	AA	GA/AA	GG	GA	AA	GA/AA	AA vs. (GA/GG)
Sex									
Male	244/383	74/116	11/21	85/137	1.00	0.99 (0.71-1.39);*P*: 0.952	0.86 (0.40-1.84);*P*: 0.705	0.98 (0.71-1.35);*P*: 0.886	0.87 (0.41-1.85);*P*: 0.713
Female	136/174	29/80	2/6	31/86	1.00	**0.45 (0.28-0.74);*P*: 0.002**	0.35 (0.07-1.79);*P*: 0.206	**0.46 (0.28-0.74);*P*: 0.001**	0.42 (0.08-2.15);*P*: 0.298
Age (years)									
<65	169/264	46/112	9/11	55/123	1.00	**0.60 (0.40-0.90);*P*: 0.013**	1.28 (0.51-3.24);*P*: 0.596	**0.67 (0.46-0.98);*P*: 0.038**	1.43 (0.57-3.60);*P*: 0.442
≥65	211/293	57/84	4/16	61/100	1.00	0.96 (0.65-1.41);*P*: 0.822	0.33 (0.11-1.03);*P*: 0.055	0.87 (0.60-1.26);*P*: 0.450	0.34 (0.11-1.04);*P*: 0.058
Smoking status									
Never	251/386	69/137	10/19	79/156	1.00	0.77 (0.55-1.07);*P*: 0.115	0.79 (0.36-1.75);*P*: 0.560	0.78 (0.57-1.07);*P*: 0.121	0.85 (0.38-1.86);*P*: 0.677
Ever	129/171	34/59	3/8	37/67	1.00	0.70 (0.43-1.16);*P*: 0.163	0.56 (0.14-2.22);*P*: 0.409	0.69 (0.43-1.12);*P*: 0.132	0.61 (0.16-2.42);*P*: 0.484
Alcohol use									
Never	345/490	94/175	9/24	103/199	1.00	0.76 (0.57-1.01);*P*: 0.061	0.53 (0.24-1.17);*P*: 0.118	0.74 (0.56-0.98);*P*: 0.035	0.57 (0.26-1.26);*P*: 0.165
Ever	35/67	9/21	4/3	13/24	1.00	0.80 (0.33-1.94);*P*: 0.620	2.28 (0.46-11.31);*P*: 0.313	0.99 (0.44-2.21);*P*: 0.976	2.37 (0.48-11.60);*P*: 0.288
BMI (kg/m^2^)									
<24	157/314	45/105	6/15	51/120	1.00	0.85 (0.57-1.26);*P*: 0.418	0.84 (0.32-2.21);*P*: 0.722	0.86 (0.59-1.26);*P*: 0.428	0.88 (0.33-2.30);*P*: 0.787
≥24	224/243	58/91	7/12	65/103	1.00	0.69 (0.47-1.01);*P*: 0.054	0.70 (0.27-1.82);*P*: 0.460	0.70 (0.49-1.00);*P*: 0.053	2.37 (0.29-1.98);*P*: 0.576

^a^ The genotyping was successful in 502 (98.80%) type 2 diabetes cases, and 782 (99.74%) controls for *PPARGC1A* rs3736265 G>A;

^b^ Adjusted for age, sex, smoking status, alcohol use and BMI (besides stratified factors accordingly) in a logistic regression model;

Bold values are statistically significant (*P* <0.05)

### SNP haplotypes

Using an expectation-maximization algorithm software [SHESIS program (Bio-X Inc., Shanghai, China, http://analysis.bio-x.cn/myAnalysis.php)] [17], we constructed thirteen haplotypes (Table 5). Haplotype comparison analysis indicated that CTTCGGG and CTCTGGG haplotypes with the order of *PPARG* rs1801282 C>G, *PPARG* rs3856806 C>T, *PPARGC1A* rs8192678 C>T, *PPARGC1A* rs2970847 C>T, *PPARGC1A* rs3736265 G>A, *PPARGC1B* rs7732671 G>C and *PPARGC1B* rs17572019 G>A polymorphisms in gene position significantly increased the risk of T2DM (OR = 1.39, 95% CI = 1.01–1.90; *P* = 0.041; and OR = 1.83, 95% CI = 1.22–2.75; *P* = 0.003, respectively). However, CCCCACA haplotype with the same order of polymorphisms in gene position conferred a decreased risk to T2DM (*P* = 0.008).

**Table 5:** *PPARG-PPARGC1A-PPARGC1B* haplotype frequencies (%) in cases and controls and risk of type 2 diabetes

Haplotypes	Cases (n=1004)	Controls (n=1564)	Crude OR (95% CI)	*P*
	n (%)	n (%)		
C C T C G G G	329(33.13)	507(32.50)	1.00	
C C C T G G G	133(13.39)	244(15.64)	0.84(0.65-1.08)	0.176
C C C C G G G	129(13.00)	200(12.82)	0.99(0.77-1.29)	0.964
C C C C A G G	98(9.82)	188(12.05)	0.80(0.61-1.06)	0.126
C T T C G G G	91(9.16)	101(6.47)	**1.39(1.01-1.90)**	**0.041**
C T C T G G G	57(5.74)	48(3.08)	**1.83(1.22-2.75)**	**0.003**
C T C C G G G	32(3.22)	72(4.62)	0.68(0.44-1.06)	0.090
C C T C G C A	31(3.12)	32(2.05)	1.49(0.89-2.49)	0.124
C T C C A G G	24(2.42)	40(2.56)	0.92(0.55-1.56)	0.770
G T T C G G G	15(1.51)	27(1.73)	0.86(0.45-1.63)	0.637
C C C C G C A	13(1.31)	16(1.03)	1.25(0.59-2.64)	0.553
G T C T G G G	11(1.11)	15(0.96)	1.13(0.51-2.49)	0.762
C C C C A C A	0 (0)	10(0.64)	-	**0.008**
Others	30(3.02)	60(3.85)	0.77(.49-1.22)	0.265

With the order of *PPARG* rs1801282 C>G, *PPARG* rs3856806 C>T, *PPARGC1A* rs8192678 C>T, *PPARGC1A* rs2970847 C>T, *PPARGC1A* rs3736265 G>A, *PPARGC1B* rs7732671 G>C and *PPARGC1B* rs17572019 G>A polymorphisms in gene position. Bold values are statistically significant (*P* < 0.05).

### Association between PPARGC1A rs3736265 G>A polymorphism and biochemistry characteristics

As pharmacotherapy for T2DM might affect biochemistry characteristics, in this stage, only non-diabetic controls were enrolled. We evaluated the association of *PPARGC1A* rs3736265 G>A polymorphism with biochemistry characteristics using Student's t-test. As shown in Table 6, there was a significant correlation of *PPARGC1A* rs3736265 G>A polymorphism with FPG. When the FPG level of *PPARGC1A* rs3736265 GG genotype was used as the reference group, FPG level of the GA/AA and GA genotypes significantly decreased [GA+AA vs. GG: *P* = 0.009 and GA vs. GG: *P* = 0.002 (Table 6)]. We also found that *PPARGC1A* rs3736265 G>A polymorphism had an association with Triglyceride. When the triglyceride level of *PPARGC1A* rs3736265 GG genotype was used as the reference group, triglyceride level of the AA genotype significantly increased (AA vs. GG: *P* = 0.014, Table 6). When the triglyceride level of *PPARGC1A* rs3736265 GG/GA genotypes was used as the reference group, triglyceride level of the AA genotype also significantly increased (AA vs. GG/GA: *P* = 0.017, Table 6).

**Table 6:** Associations of the *PPARGC1A* rs3736265 G>A genetic variants with biochemistry characteristics among control participants

Genotype	Controls (n=782)		FPG (mmol/L)	*P*	Total cholesterol (mmol/L)	*P*	Triglyceride (mmol/L)	*P*	HDL-C (mmol/L)	*P*	LDL-C (mmol/L)	*P*
	n	%										
GG	557	71.41	5.16±0.47	1.0	4.87±1.02	1.0	1.52±0.93	1.0	1.29±0.35	1.0	3.14±0.82	1.0
GA	196	25.13	**5.04±0.50**	**0.002**	4.88±1.00	0.878	1.57±0.97	0.507	1.33±0.39	0.238	3.11±0.79	0.704
AA	27	3.46	5.21±0.66	0.601	5.10±1.22	0.249	**1.97±0.95**	**0.014**	1.27±0.46	0.695	3.34±0.92	0.220
GA + AA	223	28.59	**5.06±0.53**	**0.009**	4.91±1.03	0.624	1.62±0.97	0.179	1.32±0.40	0.337	3.14±0.81	0.980
GG+GA	753	96.54	5.13±0.48	1.0	4.87±1.01	1.0	1.53±0.94	1.0	1.30±0.36	1.0	3.13±0.81	1.0
AA	27	3.46	5.21±0.66	0.395	5.10±1.22	0.249	**1.97±0.95**	**0.017**	1.27±0.46	0.606	3.34±0.92	0.197

FPG: fasting plasma glucose;

HDL-C, high-density lipoprotein cholesterol;

LDL-C, low-density lipoprotein cholesterol;

Bold values are statistically significant (*P* <0.05)

## DISCUSSION

T2DM is the most prevalent metabolic diseases worldwide, and it is multi-factorial **disorder that results from the interaction of individual's genetic background with environmental factor**. Recently, exploration of susceptibility variants has become an important approach to study the etiology of T2DM. Recent studies demonstrated that susceptibility of T2DM could be influenced by variants in some energy balance and lipid /glucose metabolism genes [18, 19]. We chose *PPARG*, *PPARGC1A*, *PPARGC1B* genes for their impact on glucose metabolism and the biological plausibility of a role in the development of IR [20, 21]. Using a case-control study approach, we investigated relationships of *PPARG*, *PPARGC1A*, *PPARGC1B* polymorphisms with T2DM. In addition, we studied the association between validated SNP and biochemistry characteristics. In this study, we identified that *PPARGC1A* rs3736265 G>A polymorphism was associated with the decreased risk of T2DM. We also found *PPARGC1A* rs3736265 A allele might modulate the level of FPG and serum triglyceride.

*PPARG* rs1801282 C>G polymorphism, a SNP in exon B, encodes a proline (Pro) to alanine (Ala) substitution at amino acid residue [22]. A previous study reported this missense substitution (Pro→Ala) might decrease transcriptional activation of *PPARG* gene *in vitro* [23]. The other important SNP in *PPARG* gene, rs3856806 C>T polymorphism, is consistently associated with higher BMI, whilst *PPARG* rs1801282 C>G polymorphism is consistently associated with a lower BMI [24]. *PPARG* rs1801282 C>G and rs3856806 C>T polymorphisms may affect the balance of energy metabolism and cell differentiation, and then presumably alter the susceptibility of T2DM. In this study, as shown in Table 3, *PPARG* rs1801282 C>G polymorphism might not confer the susceptibility to T2DM, which did not agree with results of the previous meta-analysis [25]. However, we found that only three small sample size studies with 1099 T2DM cases and 985 non-diabetic controls were included in that analysis [25]. The evidence might be limited. As for *PPARG* rs3856806 C>T polymorphism, *Du et al*. [26] and *Liu et al*. [27] found that this SNP was associated with T2DM in a Chinese population. While *Cho et al*. [28] reported a negative result in cases with gestational diabetes mellitus in the Korean population, which was similar to our results. Therefore, whether the C→T transition of rs3856806 polymorphism in *PPARG* gene does change biological activity of PPARG protein are needed to be further explored.

PPARGC1A, a transcriptional co-activator of PPARG, regulates transcription in adipogenesis, oxidative metabolism and adaptive thermogenesis relative genes [29]. Recently, some functional studies reported that PPARGC1A also control the restoration of insulin-sensitive glucose transporter (Glucose transporter type 4) gene expression in muscle cells [30], gluconeogenesis in liver and as a central target of the hepatic insulin-cAMP axis [31]. Moreover, in muscle, decreased expression of PPARGC1A was observed in diabetes cases and even in non-diabetic individuals with a family history of diabetes [32]. Several epidemiological investigations have explored the effects of *PPARGC1A* rs8192678G>A (Gly482Ser) and *PPARGC1A* rs2970847 C>T (Thr394Thr) in exon 8 and *PPARGC1A* rs3736265 G>A (Thr612Met) in exon 9 on the development of T2DM; however, the relationships have not been consistently replicated. For *PPARGC1A* rs8192678G>A polymorphism, results of some meta-analysis attained consistent findings that this SNP is associated with the increased susceptibility to T2DM in overall populations [14, 15]. While in a subgroup analysis, this association between *PPARGC1A* rs8192678G>A polymorphism and T2DM was not observed in east Asians [14], which is analogous to our findings. Previous study suggested that *PPARGC1A* rs3736265 G>A polymorphism was associated with the decreased risk of T2DM in a Danish population, and the *PPARGC1A* rs3736265A allele might be a protective factor in T2DM [33]. However, *Kim et al*. reported that *PPARGC1A* rs3736265 G>A polymorphism was not associated with the risk of T2DM in the Korean population [34]. In this study, we also focused on the association between *PPARGC1A* rs3736265 G>A polymorphism and risk of T2DM. We identified that GA/AA and GA genotype of *PPARGC1A* rs3736265 G>A polymorphism was correlated with a borderline statistically decreased susceptibility of T2DM. In subsroup analyses, we found that *PPARGC1A* rs3736265 G>A polymorphism was associated with decreased risk of T2DM in <65 years and female subgroups. As susceptibility SNP for T2DM might affect biochemistry characteristics, we also evaluated the association of *PPARGC1A* rs3736265 G>A polymorphism with biochemistry characteristics. Results of our studies indicated that *PPARGC1A* rs3736265 G>A polymorphism was associated with the decreased level of FPG, while it might increase the level of serum triglyceride. The variants of *PPARGC1A* rs3736265 G>A polymorphism decrease the level of FPG, then they might be a protective factor for the development of T2DM. These associations between *PPARGC1A* rs3736265 G>A polymorphism and biochemistry characteristics were consistent with the results of the present case-control study. However, given *PPARGC1A* rs3736265 G>A polymorphism might have opposite effects on FPG and the level of triglyceride, function of this SNP should be further explored.

A number of studies have highlighted that PPARGC1B plays an important role in regulating energy metabolism including fatty acid oxidation, thermogenesis, gluconeogenesis and mitochondrial biogenesis [13, 31, 35, 36]. The human *PPARGC1B* gene, encoding the PPARGC1B protein, locates on chromosome 5q32, a relative area that suggests linkage to T2DM [24]. The *PPARGC1B* rs7732671G>C and rs17572019G>A variants were in almost complete linkage disequilibrium in Caucasians (R^2^ = 0.958) and they were associated with the decreased risk of obesity [16]. A recent GWAS study demonstrated that *PPARGC1B* rs7732671G>C and rs17572019G>A polymorphisms were not associated with T2DM risk [12]. In this study, the distribution of genotype frequencies of *PPARGC1B* rs7732671G>C and rs17572019G>A polymorphisms was not significantly different between T2DM cases and the controls. The findings were consistent with the previous GWAS studies mentioned above.

Because *PPARG* rs1801282 C>G, *PPARG* rs3856806 C>T, *PPARGC1A* rs8192678 C>T, *PPARGC1A* rs2970847 C>T, *PPARGC1A* rs3736265 G>A, *PPARGC1B* rs7732671 G>C and *PPARGC1B* rs17572019 G>A polymorphisms may be not inherited randomly, but as construction of alleles in this study, we harnessed an online program to analyze inherited patterns of the seven SNPs. We found the frequency of CTTCGGG and CTCTGGG haplotypes with the order of *PPARG* rs1801282 C>G, *PPARG* rs3856806 C>T, *PPARGC1A* rs8192678 C>T, *PPARGC1A* rs2970847 C>T, *PPARGC1A* rs3736265 G>A, *PPARGC1B* rs7732671 G>C and *PPARGC1B* rs17572019 G>A polymorphisms in gene position was significantly increased in T2DM patients. However, CCCCACA haplotype with the same order of polymorphisms may decreased the risk of T2DM. We first reported the association of combined *PPARG*, *PPARGC1A* and *PPARGC1B* haplotypes with T2DM susceptibility.

Using an online Power and Sample Size Calculator (http://biostat.mc.vanderbilt.edu/twiki/bin/view/Main/PowerSampleSize), the power of the present study was evaluated (α=0.05). For *PPARGC1A* rs3736265 G>A polymorphism, the power was 0.469 in additive model and 0.508 in dominant model among overall T2DM group, 0.919 in additive model and 0.920 in dominant model among female subgroup, and 0.721 in additive model and 0.558 in dominant model among <65 years subgroup.

Like all epidemiological case–control studies, some limitations should be taken into account. Firstly, our study was that it was designed as a hospital-based study. All T2DM cases and non-diabetic controls were recruited from two hospitals which located in Eastern China. Although, in our study, the MAF in controls was very similar to the MAF of Chinese in the database (Table 2), the selection bias might have occurred. Secondly, we only selected seven important SNPs in *PPARG* and PPARGC1 family gene, which might not give an extensive view of the genetic susceptibility in *PPARG*, *PPARGC1A* and *PPARGC1B*. Thirdly, since significant correlation was manly found in subgroups of patients after a stratified analysis, the power of study might be limited. In the future, further studies with large sample sizes and detailed gene-environmental data are need to confirm or refute these results.

In conclusion, to the best of our knowledge, this is the first investigation about the possible correlation between *PPARGC1A* rs3736265 G>A polymorphism and biochemistry characteristics. Our findings suggest that variants of *PPARGC1A* rs3736265 G>A polymorphism decrease the level of FPG, improving the expectation of study in individual's prevention strategies to T2DM.

## MATERIALS AND METHODS

### Subjects

A total of 1,284 subjects from Eastern Chinese Han population were enrolled for this case-control study. There were 502 T2DM patients and 784 non-diabetic controls. Our study conforms to the items of the Declaration of Helsinki, and was approved by Jiangsu University (Zhenjiang, China) and Fujian Medical University Ethics Committee (Fuzhou, China). T2DM cases were selected from the department of endocrine at the Affiliated People's Hospital of Jiangsu University and the Affiliated Union Hospital of Fujian Medical University, and the controls were recruited from Health Check Centers at these hospitals. All participants provided written informed consent. All subjects were recruited between October 2014 and May 2016 consecutively. Demographic variables and risk factors of all subjects were collected by two experienced doctors. Anthropometric measurements (e.g. systolic blood pressure, diastolic blood pressure, weight and height) were tested using standard techniques. Body mass index (BMI) was assessed as weight (kilograms) divided by height (meters) squared. Serum triglycerides, total cholesterol, high-density lipoprotein cholesterol (HDL-C), high-density lipoprotein cholesterol (LDL-C), and fasting plasma glucose (FPG) were also measured. In addition, according to the criterion for overweight and obesity, a BMI of 24 was used as the cut-off point in Chinese adults [37, 38]. The information is listed in Table 1. All experimental protocol was conducted in accordance with the approved guidelines.

The World Health Organization 1999 guidelines of T2DM were used as the criteria for diagnosis [18]. For the eligible controls, the following criteria were used: no history of T2DM, postprandial plasma glucose (PPG) < 7.8 mmol/L and normoglycemia [FPG < 6.1 mmol/l)] [19].

### DNA extraction and genotyping

Samples of peripheral blood were collected with ethylenediamine tetraacetic acid (EDTA) anticoagulant vacutainer tubes (BD Franklin Lakes NJ, USA). Blood samples were stored at -20°C. Genomic DNA was extracted from lymphocytes using the Promega Genomic DNA Purification Kit (Promega, Madison, USA). The genomic DNA obtained was frozen at -80°C for SNP analysis. Genotyping of *PPARG* rs1801282 C>G, *PPARG* rs3856806 C>T, *PPARGC1A* rs8192678 C>T, *PPARGC1A* rs2970847 C>T, *PPARGC1A* rs3736265 G>A, *PPARGC1B* rs7732671 G>C and *PPARGC1B* rs17572019 G>A polymorphisms was carried out using the SNPscan^TM^ genotyping assay (Gnensky Biotechologies Inc., Shanghai, China). The success rate of all genotyping was > 99% (Table 2). The genotypes of *PPARG* rs1801282 C>G, *PPARG* rs3856806 C>T, *PPARGC1A* rs8192678 C>T, *PPARGC1A* rs2970847 C>T, *PPARGC1A* rs3736265 G>A, *PPARGC1B* rs7732671 G>C and *PPARGC1B* rs17572019 G>A polymorphisms were confirmed by the same DNA genotyping method in fifty-two (4%) randomly selected samples.

### Statistical analysis

All statistical analyses were performed in SAS 9.4 software (SAS Institute, Cary, NC). The data of continuous variables are expressed as the mean ± standard deviation (SD). Student's t-test was harnessed to determine the differences for normally distributed continuous variables between T2DM cases and controls. Chi-square test (*χ*^2^) was conducted to measure the differences for categorical variables (e.g. genotypes, sex, age, smoking status, alcohol use and BMI). We used an internet-based calculator (http://ihg.gsf.de/cgi-bin/hw/hwa1.pl) to measure the Hardy–Weinberg equilibrium (HWE) in controls with the genotype frequencies of *PPARG* rs1801282 C>G, *PPARG* rs3856806 C>T, *PPARGC1A* rs8192678 C>T, *PPARGC1A* rs2970847 C>T, *PPARGC1A* rs3736265 G>A, *PPARGC1B* rs7732671 G>C and *PPARGC1B* rs17572019 G>A polymorphisms. The associations between *PPARG* rs1801282 C>G, *PPARG* rs3856806 C>T, *PPARGC1A* rs8192678 C>T, *PPARGC1A* rs2970847 C>T, *PPARGC1A* rs3736265 G>A, *PPARGC1B* rs7732671 G>C and *PPARGC1B* rs17572019 G>A polymorphisms and risk of T2DM were assessed by crude ORs and adjusted ORs when it was appropriate. SHESIS software (Bio-X Inc., Shanghai, China, http://analysis.bio-x.cn/myAnalysis.php) was used for construction of haplotypes [17]. A *P* < 0.05 (two-tailed) was considered as the criterion of statistical significance.
